# Supplementary material for: Perinatal outcome of vaginal breech delivery in Orotta National Referral Teaching Hospital, Eritrea, 2024; a case control study
Source: PLoS One. 2025 Oct 6;20(10):e0317262. doi: 10.1371/journal.pone.0317262 (PMC12500088; doi:10.1371/journal.pone.0317262)
Supplement: S1 Checklist — (PDF) [file pone.0317262.s001.pdf]

Human Participants Research Checklist

Complete the following if your study involved human participants or human participants' data. These questions should be addressed for prospective and retrospective studies.

1. Did you obtain ethics approval for this study?
    - If yes, please upload (file type "Other") the original approval document you received from your ethics committee. <u>If the original document is in another language, please also provide an English translation.</u>

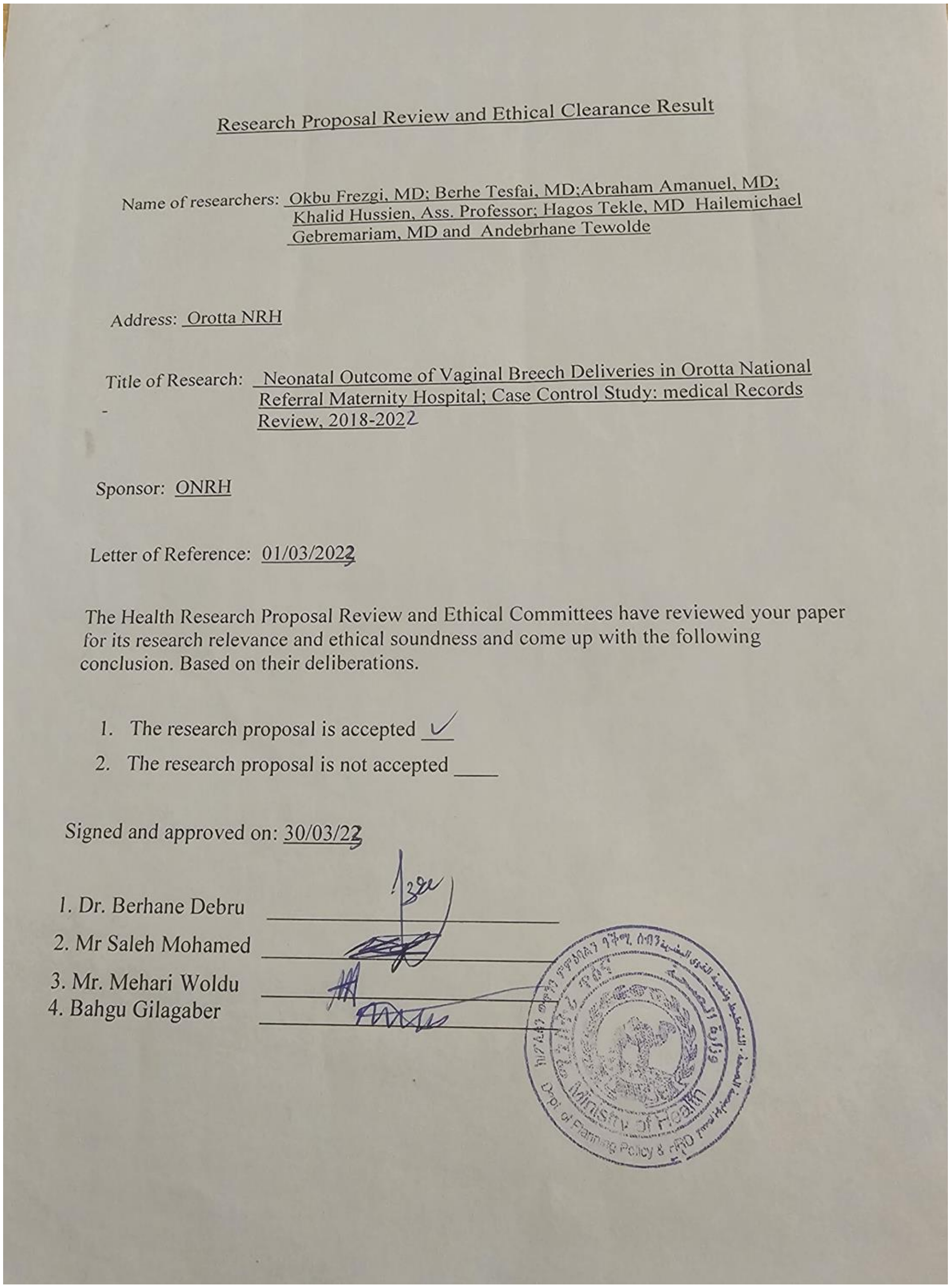

Research Proposal Review and Ethical Clearance Result

Name of researchers: Okbu Frezgi, MD; Berhe Tesfai, MD;Abraham Amanuel, MD; Khalid Hussien, Ass. Professor; Hagos Tekle, MD Hailemichael Gebremariam, MD and Andebrhane Tewolde

Address: Orotta NRH

Title of Research: Neonatal Outcome of Vaginal Breech Deliveries in Orotta National Referral Maternity Hospital; Case Control Study: medical Records Review, 2018-2022

Sponsor: ONRH

Letter of Reference: 01/03/2023

The Health Research Proposal Review and Ethical Committees have reviewed your paper for its research relevance and ethical soundness and come up with the following conclusion. Based on their deliberations.

1. The research proposal is accepted ✓
2. The research proposal is not accepted ____

Signed and approved on: 30/03/23

1. Dr. Berhane Debru ____________
2. Mr Saleh Mohamed ____________
3. Mr. Mehari Woldu ____________
4. Bahgu Gilagaber ____________

✓ Uploaded ___ N/A

- If you did not obtain ethical approval, please explain why this was not required below.

2. If you prospectively recruited human participants for the study – for example, you conducted a clinical trial, distributed questionnaires, or obtained tissues, data or samples for the purposes of this study, please report in the Methods:
   i. the day, month and year of the **start and end** of the recruitment period for this study.
   ii. whether participants provided informed consent, and if so, what type was obtained (for instance, written or verbal, and if verbal, how it was documented and witnessed). If your study included minors, state whether you obtained consent from parents or guardians. If the need for consent was waived by the ethics committee, please include this information.

___ Completed __✓_ N/A

3. If you are reporting a retrospective study of medical records or archived samples, please report in the Methods section:
   i. the day, month and year when the data were accessed for research purposes
   ii. whether authors had access to information that could identify individual participants during or after data collection

_✓__ Completed ___ N/A

the day, month and year when the data were accessed for research purposes the day, month and year when the data were accessed for research purposes
