# Supplementary material for: Perinatal outcome of vaginal breech delivery in Orotta National Referral Teaching Hospital, Eritrea, 2024; a case control study
Source: PLoS One. 2025 Oct 6;20(10):e0317262. doi: 10.1371/journal.pone.0317262 (PMC12500088; doi:10.1371/journal.pone.0317262)
Supplement: Attachment — Submitted filename: Authors respones final.docx [file pone.0317262.s003.docx]

| **Reviewers comments** | **Authors response** |
| --- | --- |
| **Reviewer #1** | |
| Research question is not clearly answered | Thank you  The main aim of this study was to identify the perinatal outcome in vaginal breech deliveries and we have got the results including low first minute Apgar. |
| A couple of results mentioned in Discussion [200-1] do not Appear in Results | Correction was made as these variable were significant only in bivariate analysis |
| Use of perinatal vs. neonatal | Thank you, we preferred perinatal and arrangements were made |
| uniformity of CD vs. CS | We preferred CS and uniformity was made. |
| provide definitions of abbreviations (COR, AOR) | Acronym provided |
| what is VCD [100] | It means vertex vaginal delivery. We changed it to cephalic vaginal delivery. |
| univariable vs. univariate | Changed to univariate |
| provide definitions of abortion and extremely premature | Operational definitions given for abortion but giving definition for extreme prematurity is above the scope of this paper. It is not important as we didn’t identify the gestational age during delivery |
| risk vs. likelihood | We preferred risk |
| Difficult to interpret in places (especially multivariate analyses - unclear what is being isolated to highlight effect of exposure of interest) | Thank you very much, arrangements were made.  The variables that were found non-significant association with the outcome were omitted from further analysis. The multivariable analysis included only those variables that were significantly associated with the outcome variable univariate analysis. The association was considered significant if p-value is <=0.05. |
| No sample size calculation | There was no sample size calculation. Initially we considered calculating the sample size however, the study assumed using newly introduced or updated data recording system that was introduced in 2019. This option provided us a chance to involve more participants in the study rather than the sample size that could be provided by the formula. Moreover, involving more events in the study did not incur us extra cost. |
| Unclear why 1:2 ratio of case: control | The choice of 1:2 ratio of cases to controls was based on the idea that it was easy to recruit cases and controls with no extra cost, and the outcome was considered more common event. |
| Median is mentioned (how is it relevant) | It is omitted, only mean was measured to represent central tendency for continuous variables. |
| Mean should be presented with SD | Thank you correction have been made  Mean mothers age 29.89, SD=5.56  Mean parity 2.18, SD=1.96 |
| it may have been better to exclude multiple pregnancies | As we are assessing the perinatal outcome which is equally important for both singleton and multiple fetuses we decided to include all deliveries. If the aim of the study was about the success of VBD we would exclude them. |
| I think the ethics statement in the proforma should refer to the approval by the Research Ethics committee | The study strictly adhered to ethical guidelines and Ethical approval was obtained from the Ministry of Health Research Approval and Ethical Committee (reference number 30/03/2023). It refers to the Research Ethics committee |
| Unclear if maternal morbidity referred to in [64] is immediate vs. long-term. Also unclear what the nature of maternal morbidity related to VBD is | This was in literature review and they didn’t specify whether it’s long term or short term. We didn’t assess maternal outcome. |
| What is your reason for using the birth strata you used | To substantiate our observational view of birth asphyxia related to VBD |
| Can you comment on the significance of the decrease in VBD over the study period, and the potential reason | In the hospital a residence program in Obstetrics and gynecology reinstituted in 2019 and the rate caesarean section increased and this could be the main reason |
| Citations - Many listed as invalid | Arrangements were made |
| Limitations - I feel there are more than are mentioned | All the limitation of the study were included |
| **Reviewer #1** | |
| The results need to be noted | Thank you, the results were completely analyzed again and extensive arrangements were made |
| What I missed it the fact whether the breech deliveries ("cases") had a fetal heart on admission in labor or not. i.e. Were all fresh stillbirths. That should be confirmed and discussed at the relevant positions | This was reflected on limitation of the study. We couldn’t identify fetal status from the records. |
| In the discussion I also missed the argument that the increased rate of stillbirth may be related to the relatively poor experience of vaginal breech deliveries of the current clinicians? | This issue was discussed in [196-199] that poor intrapartum care and intrapartum identification of breech presentation might increase the stillbirth. |
