# Supplementary material for: Perinatal outcome of vaginal breech delivery in Orotta National Referral Teaching Hospital, Eritrea, 2024; a case control study
Source: PLoS One. 2025 Oct 6;20(10):e0317262. doi: 10.1371/journal.pone.0317262 (PMC12500088; doi:10.1371/journal.pone.0317262)
Supplement: Attachment — Submitted filename: Authors respones final 16-07-25.docx [file pone.0317262.s004.docx]

| **Reviewers comments** | **Authors response** |
| --- | --- |
| **Reviewer #1** | |
| 1. See lines 85/86. As previously suggested, use perinatal OR neonatal. Title now says Perinatal, but neonatal is used twice in these lines. | Thank you. Arrangements have being made. |
| 2. Choose either multivariable or multivariate <https://pmc.ncbi.nlm.nih.gov/articles/PMC3518362/> | Thank you. Arrangements have being made. |
| 3. Suggest listing a lack of a sample size calculation as a limitation. Same with decision to use 1:2 case: control. Your argument makes logical sense, but we are not sure if it makes statistical sense. | Added in limitation part with explanation.  But, the choice of 1:2 ratio of cases to controls was based on the idea that it was easy to recruit cases and controls with no extra cost, and the outcome was considered more common event. For this reason we didn’t add in limitation part. |
| 4. Median is still mentioned (line 110) | Arrangements have being made. |
